# The Behavior of Terbuthylazine, Tebuconazole, and Alachlor during Denitrification Process

**DOI:** 10.3390/jox13040036

**Published:** 2023-10-01

**Authors:** Kristína Pániková, Zuzana Bílková, Jitka Malá

**Affiliations:** 1 Institute of Chemistry, Faculty of Civil Engineering, Brno University of Technology, 602 00 Brno, Czech Republic; mala.j@fce.vutbr.cz; 2 Research Centre for Toxic Compounds in the Environment, Faculty of Science, Masaryk University, 611 37 Brno, Czech Republic

**Keywords:** alachlor, terbuthylazine, tebuconazole, inhibition, laboratory denitrification assay

## Abstract

Pesticide compounds can influence denitrification processes in groundwater in many ways. This study observed behavior of three selected pesticides under denitrifying conditions. Alachlor, terbuthylazine, and tebuconazole, in a concentration of 0.1 mL L^−1^, were examined using two laboratory denitrifications assays: a “short” 7-day and a “long” 28-day test. During these tests, removal of pesticides via adsorption and biotic decomposition, as well as the efficiency of nitrate removal in the presence of the pesticides, were measured. No considerable inhibition of the denitrification process was observed for any of the pesticides. On the contrary, significant stimulation was observed after 21 days for alachlor (49%) and after seven days for terbuthylazine (40%) and tebuconazole (36%). Adsorption was in progress only during the first seven days in the case of all tested pesticides and increased only negligibly afterwards. Immediate adsorption of terbuthylazine was probably influenced by the mercuric chloride inhibitor. A biotic loss of 4% was measured only in the case of alachlor.

## 1. Introduction

Pesticide compounds are used to protect crops against pests and diseases; on the other hand, they are one of the most noxious environmental pollutants due to their mobility, stability, accumulative and persistent nature, and adverse chronic effects on living organisms. More than 3.5 × 10^6^ t of pesticides were used worldwide in 2021, 3461 t out of which were applied to agricultural land in the Czech Republic [1]. According to the European Commission [2], there are 1464 types of active pesticide substances; among them, 454 are approved, 927 are not approved, and 17 are under review. Mixtures of two or more pesticides often react and/or form complex substances that may express properties unique to that combination [3]. Nevertheless, in many areas, several pesticides are used simultaneously on agricultural crops, which leads to a higher risk and increased pollution [4].

Pesticides are primarily applied to agricultural land, but these substances can be transported over long distances through groundwater and surface water by flow, leaching, and pulverization processes. They can also reach water bodies by surface runoff and by percolation through the soil into the groundwater [3]. According to expert estimates, 10% of all pesticides applied to the soil reach non-target areas [5]; however, this estimated value is also affected by weather [3]. Apart from rainfall, groundwater contamination is related to the frequency of pesticide application and the intensity of spraying, as well as to the properties of the soil and aquifer recharge rate [6]. 

Pesticides often decompose, undergo various transformations, and come into contact with other substances and metabolites. In water, they may metamorphose to produce substances with even greater toxicity than parent compounds [7]. When pesticides reach water bodies, they can impact the whole ecological food chain, including humans [3]. Therefore, the presence of pesticides and their metabolites in groundwater represents a potential health risk for humans and thus presents a serious environmental issue [8], especially in communities dependent on groundwater as a major source of drinking water.

In Europe, more than 65% of the drinking water sources are groundwaters [8]. However, many European groundwater bodies are polluted by pesticides, mainly atrazine and terbuthylazine and their desethyl-degradation metabolites, at concentrations higher than 0.1 μg L^−1^ [8]. Pesticide residues are also commonly found in waters throughout the Czech Republic. For more than 9 and 13 years, respectively, alachlor, acetochlor, and atrazine have been banned in the Czech Republic; however, these compounds are still being detected in groundwaters [9]. Studies have also detected the metabolites of chloridazon, metazachlor, metolachlor, glyphosate, and dimethachlor in groundwaters [9]. Consequently, about 70% of tap water supply networks are affected by pesticide contamination, which is much more than previously thought. In more than 5% of the cases, set limit values for these substances are exceeded. Moreover, since 2017, pesticides have been the most common cause of “exceptions” to drinking water quality requirements [10]. 

Because of the increasing number of contaminated drinking water sources, it is necessary to understand the behavior of pesticides in anoxic conditions and to develop methods capable of removing them from groundwater [8]. Removal of pesticides can be accomplished via both biotic and abiotic processes [8,11]. According to Fenner et al. [11], a pesticide undergoes transformation processes, the type of which is determined by its structure, distribution, transport behavior, and by environmental conditions. Unfortunately, predicting the behavior of pesticides under denitrifying conditions typical for groundwater is both a scientific and a practical issue [12]. According to Chen et al. [13], the addition of carbon can support the removal efficiency of pesticides in groundwater. Nevertheless, as yet, there is very little other information available. In fact, knowledge about the behavior of pesticides under denitrification conditions is, in general quite limited, even for the most common pesticide substances. 

Pesticides are often applied on agricultural land together with other chemical substances, e.g., fertilizers. Between 1960 and 1990, a high increase in nitrogen use in agriculture was recorded; over half of the synthetic nitrogen fertilizers produced had been used between the years 1993 and 2008 [14]. Excess nitrogen due to over-fertilization can leach into groundwater through the unsaturated zone [15]. Nitrates are a very mobile form of nitrogen in soil, representing a dangerous pollutant of waters, and may cause many diseases [16]. Thus, groundwater nitrate–nitrogen contamination has become a considerable environmental problem and potential risk for human health [17]. Nevertheless, under anoxic conditions, typical for groundwater, nitrates and nitrites can be reduced via heterotrophic biological denitrification to elemental nitrogen or nitrogen oxides, which do not pose substantial risk to the environment. The effects of pesticides on the denitrification process are largely unknown [18]. Michel et al. [19] have strongly insisted on the need to carry out studies at low pesticide/metabolite concentrations to obtain a realistic picture on how these molecules can affect subsurface microbial communities and activities.

This study investigated the behavior of three pesticides (tebuconazole, terbuthylazine, alachlor) during the process of denitrification using laboratory denitrification assays, which were developed and optimized by the authors [20]. Specifically, the aim was to gain knowledge of pesticide biodegradation and adsorption on organic substrate, as well as the effects of these compounds on the denitrification process.

Since atrazine was banned in the European Union, terbuthylazine (TER) has become a common choice instead. Terbuthylazine is ranked as one of the most persistent triazine herbicides in terrestrial and aqueous environments [21] and is very persistent in soil, plants, and animals. 

Alachlor (ALA) is a chloroacetanilide herbicide. It was used in the mid-1990s and subsequently replaced by acetochlor because of its carcinogenity [22]. This herbicide is relatively stable in water, can be detected in groundwater years after its application to the soil, and migrates with groundwater away from areas of use [23]. 

To control the presence of fungi, a systemic fungicide tebuconazole (TEB) is commonly applied on vegetables and fruits [6]. This pesticide has toxic effects on non-target organisms [24], is persistent in soils, and presents low-to-moderate mobility [25]. It is intensively adsorbed by soils and is mainly captured in the topsoil layer [26].

## 2. Materials and Methods

The selected pesticides (TEB, TER, ALA) were tested using both seven-day batch denitrification assays (“short test”, ST) and 28-day semi-continuous denitrification assays (“long test”, LT). Laboratory tests were performed in the same way as in Pániková et al. [20]. 

### 2.1. Principles of the Assays

In the assays, the denitrification rates and the concentrations of the tested contaminants in three different treatments are compared (Table 1):

- Control treatment (C): Denitrification is in progress without disturbance;
- Treatment 1: Denitrification process is affected by the presence of the tested pesticide; at the same time, the conditions for both biotic and abiotic loss of the tested pesticide are created;
- Treatment 2: Biological processes (denitrification and biotic loss of the tested pesticide) are stopped, while abiotic loss of the tested pesticide is in progress.

The effect of the tested pesticide on the denitrification INH(den) is assessed via the difference of the denitrification rates r_D_ in treatments C and 1, with positive values for inhibition and negative for stimulation, as follows:

(1) $$\mathrm{INH}\left(\mathrm{den}\right)=\frac{r_{D}\left(C\right)-{\;r}_{D}\left(1\right)}{r_{D}\left(C\right)}\times 100\;\left[\%\right]$$

(2) $$r_{D}=\frac{c{\left({\mathrm{NO}}_{X}-N\right)}_{\mathrm{init}}-\;c{\left({\mathrm{NO}}_{X}-N\right)}_{\mathrm{end}}}{n}\;\left[{\mathrm{mg}\;L}^{-1}{\;d}^{-1}\right],$$

where c(NO_X_-N)_init_ is the initial concentration of nitrate-and-nitrite nitrogen [mg L^−1^], c(NO_X_-N)_end_ is its final concentration [mg L^−1^], and n is the duration of the assay [days]. 

The loss of the tested pesticide in treatment 1 (D_tot_) corresponds to the total loss caused by both biotic and abiotic processes:

(3) $$D_{\mathrm{tot}}=c_{1\;\mathrm{init}}-c_{1\;\mathrm{end}\;}\;\left[{µg\;L}^{-1}\right],$$

where c_1init_ and c_1end_ are the initial and final concentrations of the tested pesticide in treatment 1 [µg L^−1^]. The abiotic loss of the tested pesticide (D_abio_) is calculated from treatment 2:

(4) $$D_{\mathrm{abio}}=c_{2\;\mathrm{init}}-c_{2\;\mathrm{end}\;}\left[{µg\;L}^{-1}\right],$$

where c_2init_ and c_2end_ are the initial and final concentrations of the tested xenobiotic in treatment 2 [µg L^−1^]. The biotic loss (D_bio_) is expressed as follows:

(5) $$D_{\mathrm{bio}}=D_{\mathrm{tot}}-D_{\mathrm{abio}}\;\left[{µg\;L}^{-1}\right].\;$$

### 2.2. Chemicals and Organic Carrier

Terbuthylazine, tebuconazole, and alachlor (PESTANAL^®^ product line) were obtained from Sigma-Aldrich (Taufkirchen, Germany) at a purity higher than 98%. Pesticide stock solutions were prepared in analytical-grade methanol at a concentration of 1000 mg L^−1^ and stored in the dark at 4 °C. 

Wood shavings were used as a bacterial carrier and a source of organic carbon. The wood shavings were obtained from poplar trees growing in the territory of the Slovak Republic. After cutting the trees to shavings, these were sieved at the 1.0–1.5 cm fraction. 

Argon gas, at the purity of 99.996%, was obtained from Linde Gas (Prague, Czech Republic).

### 2.3. Analytical Methods 

The laboratory analyses were performed in the following manner: dissolved oxygen (DO) and pH were measured with a Hach HQ40D multi-meter (Hach Lange GmBH, Düsseldorf, Germany), chemical oxygen demand (COD) was determined via the semi-micro method with potassium dichromate, and photometric evaluation was assessed at 445 nm using a DR3900 spectrophotometer (Hach Lange GmBH, Düsseldorf, Germany; ISO 8192:2007), NO_X_-N (NO_3_-N+NO_2_-N) was assessed via the UV absorption method (Harris, 2003) with a Hach optical Nitratax plus sc Sensor (Hach Lange GmBH, Düsseldorf, Germany), and NO_2_-N was assessed via the photometric method with sulphanilic acid and 1-naphtylamine at 515 nm [27] using a DR3900 spectrophotometer (Hach Lange GmBH, Düsseldorf, Germany).

Solid-phase extraction (SPE) was used to extract pesticides from the water samples to cartridges. The cartridges (Oasis HLB, 6 mL, 0.5 g HLB sorbent material) (Waters, Milford, MA, USA) were activated with 7.5 mL of methanol:acetone (3:2) (Sigma-Aldrich, Steinheim, Germany) and washed with 7.5 mL of mili-q water. A 5 mL water sample and 100 µL of internal standard (IS) were passed through the SPE cartridge. Metolachlor (c = 5 µg mL^−1^) was used as IS for terbuthylazine and tebuconazole. Epoxikonazole (c = 5 µg mL^−1^) was used as IS for alachlor. Subsequently, the SPE cartridges were washed with 7.5 mL of deionised water and then air-dried for 5 min. The adsorbed pesticides were eluted with 5 mL of methanol:acetone (3:2). High-performance liquid chromatography (HPLC) analysis was performed using an Agilent 1200 chromatographic system (Agilent, Santa Clara, CA, USA) equipped with an Agilent Triple Quad 6410 mass spectrometer (Agilent, Santa Clara, CA, USA). The mean water recoveries of pesticides were 98%, and the limit of quantification (LOQ) achieved in the water samples was 1 µg L^−1^.

## 3. Results and Discussion

### 3.1. Conditions of Laboratory Denitrification Assays

Measured pH in all tests reached values between 7 and 8, i.e., in the optimal range for denitrification [28,29]. 

At the end of all ST assays, low values of DO (<0.5 mg L^−1^) were found. This was optimal for the course of denitrification, which requires low or no concentrations of DO (<0.5 mg L^−1^), i.e., anoxic conditions [30]. However, after 28 days, average DO range from 0.91 mg L^−1^ (TEB) to 2.50 mg L^−1^ (TER) in both treatments C and 1. Similar results were observed in a previous study with the metolachlor pesticide [20]. However, it can be assumed that denitrification processes continued under anoxic conditions, probably prevailing in micropores of the organic carrier. Therefore, denitrification took place even with increased DO in water, as can be seen from Table 2.

In ST assays with ALA and TEB, average values of NO_2_-N were high in both control (C) and treatment 1. For the control treatment, the differences between parallel replicates were large, as shown by the standard deviations; two replicates had low values and two very high ones, these results thus cannot be unequivocally evaluated. Treatment 1 also showed a high standard deviation; however, all replicates had NO_2_-N concentrations higher than 3 mg L^−1^. This suggests that in this treatment, the first step of the denitrification took place—nitrates were reduced to nitrites—but the reduction to N_2_O did not continue, similarly to that which was observed by Hu et al. [31]. According to Qian et al. [32], accumulation of nitrites during the denitrification process can be caused by higher pH values. However, this is not in accordance with our results because in all cases, pH was in the optimal range and DO was low. Nevertheless, Chung and Bae [33] state that nitrite reduction is more sensitive to pH changes than nitrate reduction. They reported that the nitrite reduction rate fell sharply at a pH lower than that which the tested denitrifying bacteria had been acclimated to, while the nitrate reduction rate remained unchanged. We assume that this effect could explain the results in our ST assays. 

After 28 days, in the LT assays, lower concentrations of nitrites were found in all cases, suggesting that they were reduced to N_2_O or N_2_, meaning that the process of denitrification continued without disturbance.

Since an organic substrate is necessary for denitrification as it is oxidized during the process, acting as the source of energy [29], COD was measured at the end of all tests. According to Lahdhiri et al. [34], the minimum COD/N ratio for complete denitrification lies between 3.5 and 5. In all our samples, a ratio of the residual COD after the denitrification and the initial concentration of NO_3_ between 6.8 and 9.7 was obtained, showing that the measured concentrations of organic compounds were sufficient to foster the denitrification process [15,34]. 

### 3.2. Inhibition of Denitrification Process

**Figure 1:**
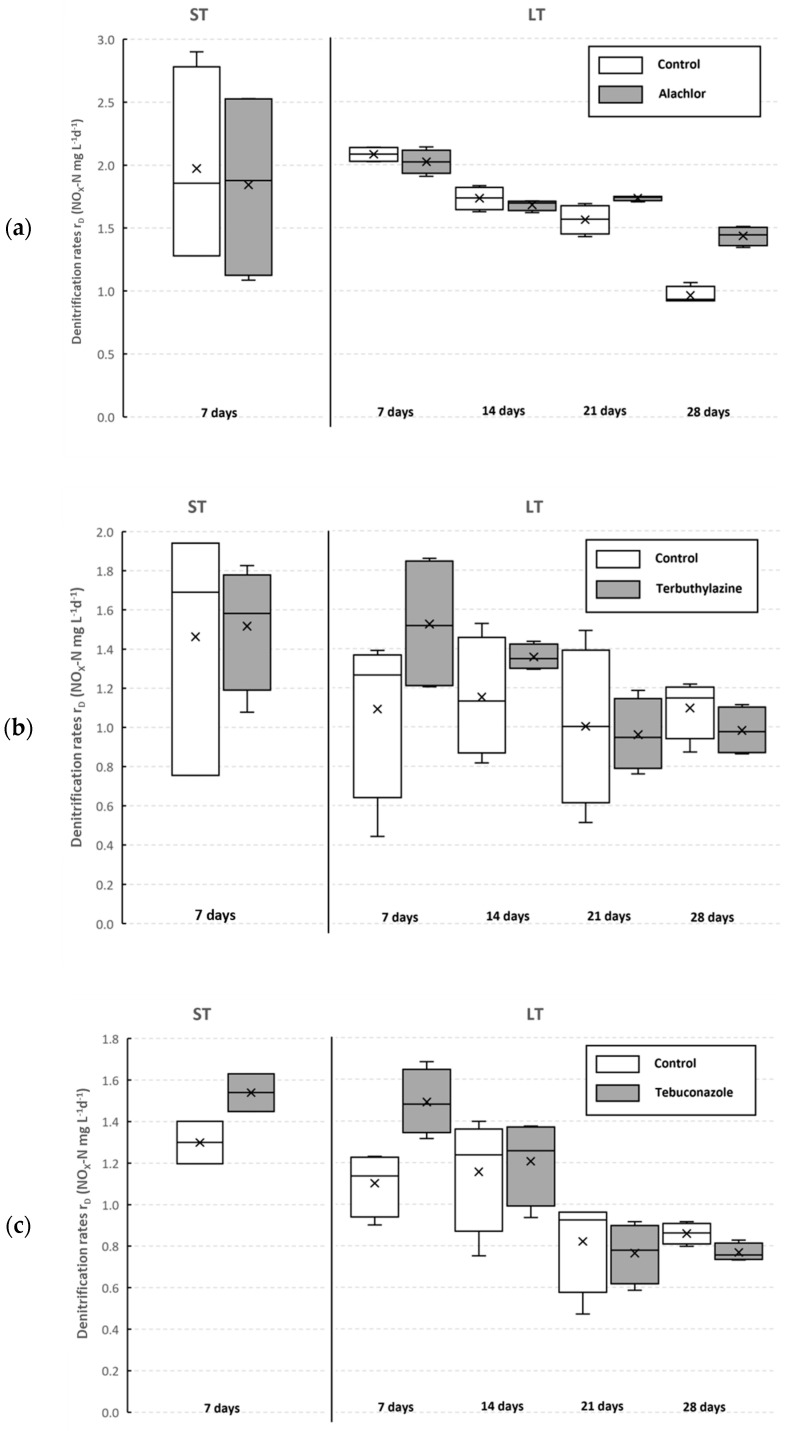
Denitrification rates (mean, median, min, max) in the batch assays for alachlor (**a**), terbuthylazine (**b**), and tebuconazole (**c**).

For all pesticide compounds tested, denitrification rates observed in the treatments without the tested pesticide (control) and with the pesticide (treatment 1) were compared (Figure 1). No inhibition or significant stimulation of the denitrification process was found for any of the tested pesticides, with the exception of ALA in the LT assay.

Denitrification rates observed in the ST assay with ALA were moderately higher in the control (1.97 mg L^−1^ d^−1^) than in the treatment with ALA (1.84 mg L^−1^ d^−1^), which means that inhibition of 6.6% was measured (Figure 1a). Similar results were measured after the first and the second week of the LT; negligible inhibition of 2.8% after the first 7 days increased only slightly (to 3.0%) after the second week. However, a significant stimulation of the process was observed afterwards—10.8% after 21 days, and more than 49% at the end of the incubation—which corresponded with denitrification rates of 0.96 ± 0.06 (control) and 1.43 ± 0.07 mg L^−1^ d^−1^ (treatment 1). 

Based on these results, it seems that after an initial period of ca. 14 days, ALA significantly fostered the denitrification process. This finding supports the study of Novak et al. [35], who did not find any inhibition of the denitrification process in the presence of ALA. Pozo et al. [36] studied the effects of the ALA pesticide in a soil environment at a dosage of 2.0–10.0 kg ha^−1^ on, among other things, bacterial and fungi populations, and found that the presence of ALA in the soil increased the total number of bacteria and fungi. The population of denitrifying bacteria increased significantly at concentrations above 5.0 kg ha^−1^. Therefore, an increase in abundance of denitrifying bacteria could be the reason for the stimulation of the denitrification rate in our experiments as well. To confirm our observations, further experiments with this pesticide should be performed, ideally with more than four replicates. 

A comparison between the control and treatment 1 with TER showed negligible stimulation of 3.7% in the ST assay (Figure 1b). After the first seven days of LT assay, the stimulation was much higher (39.8%). However, over the next 3 weeks, the stimulation effect decreased (Figure 1b), and at the end of the assay, an inhibition was observed, namely, 10.5%. These results could not be compared with those from previous studies since no information regarding inhibition or stimulation of the denitrification by TER was found in the relevant literature.

Similar results were observed in the case of the TEB pesticide (Figure 1c). Its stimulation effect reached the values of 18.5% and 35.5% after the first 7 days of the ST and LT assay, respectively. During the next 3 weeks, this effect gradually decreased, as in the case with TER, and at the end of the LT assay, an inhibition of 10.6% was measured. 

Cycoń et al. [37] observed that numbers of heterotrophic bacteria were strongly stimulated with TEB in sandy loam soil. During 28 days of their experiments, cultivable denitrifying bacteria were not affected by the addition of TEB at doses > 2.7 mg kg^−1^; however, increased stimulation was measured on day 14 and, to a lesser extent, on day 28 of these experiments. Cycoń et al. [37] claim that the inhibition of denitrification by TEB can be associated with increased nitrate concentrations. This could explain the gradual shift from stimulation to inhibition observed in our results since, during the LT assay, nitrate was added every week. 

### 3.3. Removal of the Pesticides during Denitrification Process

**Figure 2:**
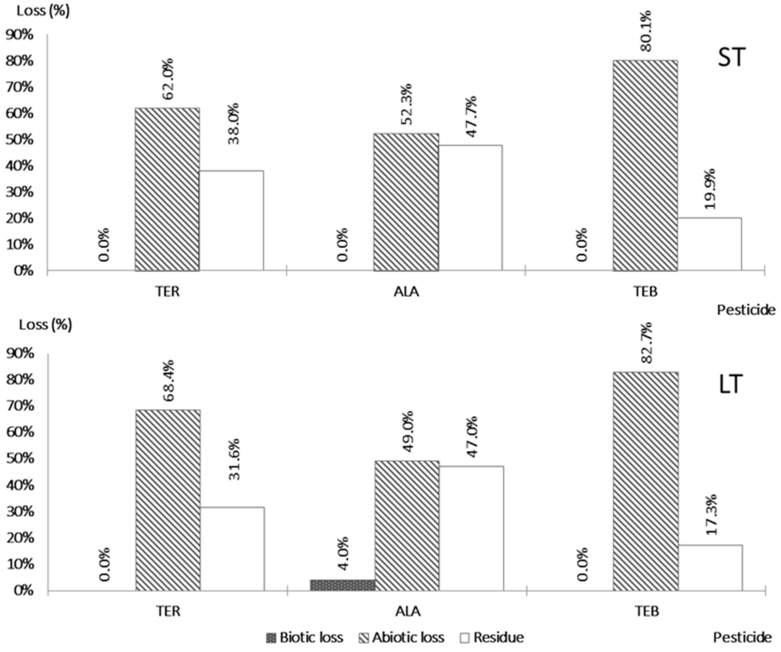
Proportions of individual pesticides decomposed by biotic/abiotic processes or remaining as residue after the assay ends.

The tested pesticides are different in terms of their chemical composition. A triazine pesticide TER is 2-N-tert-butyl-6-chloro-4-N-ethyl-1,3,5-triazine-2,4-diamine. A chloroacetanilide herbicide ALA is 2-chloro-N-(2,6-diethylphenyl)-N-(methoxymethyl)acetamide. In addition, a systemic fungicide TEB is 1-(4-chlorophenyl)-4,4-dimethyl-3-(1,2,4-triazol-1-ylmethyl)pentan-3-ol. Accordingly, the results, i.e., the fate of the pesticide during the denitrification process, differed for the individual pesticide substances (Figure 2).

No biotic loss was found for TER and TEB after both 7 and 28 days. This was not surprising in the case of TER, considering the findings of Navarro et al. [12], who determined the dissipation half-life of TER in groundwater to range from 263 to 366 d. Since TER is considered the most persistent triazine herbicide in surface environments [21], a 28-day test is not long enough for the assessment of biotic degradation under the denitrifying conditions. However, in the case of TEB, some biotic loss was expected since Caldas et al. [6] reported the half-life of TEB in groundwater to range between 7 and 28 d. 

In the case of ALA, a negligible biotic loss of 4.0% was found after 28 days. These results are in accordance with those of Graham et al. [38], who researched the degradation of ALA in aquatic mesocosm under variable oxygen conditions and observed the shortest half-life, namely, 9.7 days, in anaerobic conditions. Colosio et al. [23] observed a half-life of 8 days in a soil environment. Dehghani et al. [39] studied the effects of carbon sources on ALA biodegradation. Different organic compounds, such as glucose, sodium citrate, sucrose, and starch, and also the combination of these carbon sources, were tested in this study. The availability of the carbon source was found to foster the ALA dissipation rate, as the sample without a carbon source had the lowest degradation rate. The results of our study further suggest that organic carbon released from poplar wood shavings can support the degradation.

**Figure 3:**
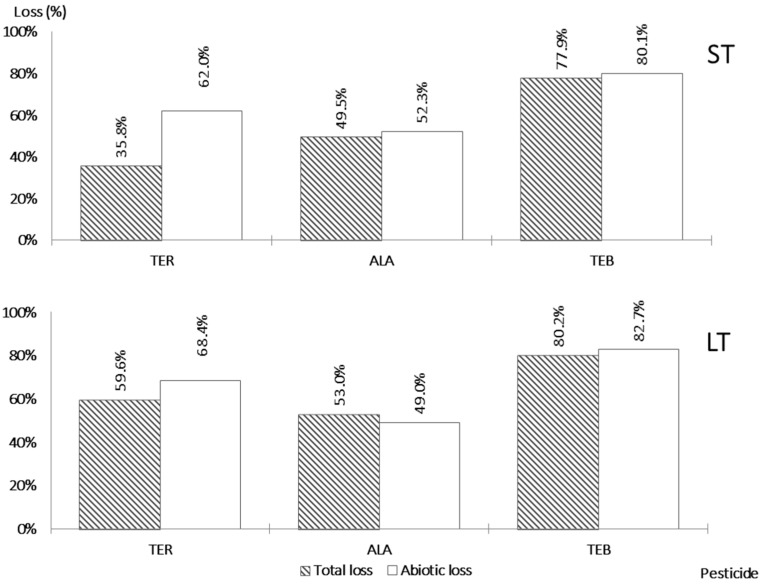
Comparison of the losses in treatment 1 and 2.

The adsorption was in progress at the beginning of the incubation and increased only negligibly afterwards in the case of all three pesticides (Figure 3). 

TEB showed the highest abiotic loss of all tested pesticides after 7 days, namely, 80.1%. Abiotic loss in LT was only slightly increased (82.7%). Čadková et al. [40] also observed the high adsorption of TEB in soils containing high proportions of organic matter. In contrast, Caldas et al. [6] observed a total loss of TEB in groundwater of only around 50% after 7 days; this study was carried out in an agricultural area and samples were taken from drinking water wells. Herrero-Hernández et al. [26] stated that the removal of TEB is affected by leachable organic carbon content in the adsorbent, with high contents of organic carbon contributing to increased adsorption. Similarly, in the studies of Ilhan et al. [41] and Krause Camilo [42], it was also observed that the main part of abiotic loss was associated with the sorption of pesticides to the wood shavings. These studies analyzed the potential of organic substrates to retain both nitrate and the agrochemicals using the technology of denitrification bioreactors. All the above findings are in accordance with the presented results since, in our assays, the poplar shavings acted as an organic adsorbent.

An abiotic loss of ALA of 52.3% was measured after 7 days. Abiotic loss after 28 days in the LT assay was similar, namely, 49.0%. These results show that adsorption was in progress during the first 7 days and did not continue afterwards. Ahmad [43] observed that the adsorption capacity for ALA varied in soils depending on their physicochemical properties, and the highest adsorption–desorption distribution coefficient K_d_ was found in the soil sample with the highest organic content. In his study, activated carbon from sawdust was investigated as an adsorbent, and the highest removal percentage was around 64% after 48 h. This is in accordance with our results regarding the adsorption.

With TER, we measured abiotic losses of 62.0% in the ST assay and 68.4% in the LT assay. These results could not be compared with those from previous studies, since any information regarding the adsorption of TER was found in the relevant literature.

The methodology used assumes that the dissipation of the pesticides will be comparable or lower in treatment 2, with the addition of the HgCl_2_ inhibitor, than in treatment 1, where only the pesticide is present, i.e., both biotic and abiotic losses can occur. This was the case for ALA and TEB, for which, at the end of both ST and LT tests, the losses were comparable (Figure 3). However, in the case of TER in ST, the loss for treatment 1 (35.8%) was only half that of treatment 2 (62.0%), although the difference was lower after 28 days (Figure 3). Since the only major difference between treatment 1 and 2 was the content of HgCl_2_, these results suggest that immediate adsorption was probably influenced by this compound. The results of abiotic loss regarding this pesticide, therefore, cannot be taken into consideration. This behavior has not been observed before and should be analyzed in more detail in further studies.

Although some biotic loss was measured in the case with ALA, this pesticide had higher values of residue, namely, 47.7% after seven days and 47.0% after 28 days. The highest decrease in residue was found for TER, where the change was from 38.0% to 31.6%. In both tests with TEB, the lowest values of the mean residues were observed (19.9% and 17.3%). 

Depending on the persistency of pesticide compounds, their half-life may be a few hours/days or, for persistent ones, more than a year. It is possible that for some substances, e.g., TER, the degradation would accelerate after the initial lag phase. Twenty-eight days could be a short time for the decomposition of such persistent pesticides; however, it is difficult to sustain denitrifying conditions for longer periods under laboratory conditions. Although we have not observed any inhibition of the denitrification process, it is necessary to take into consideration the possible transformation of parent pesticides to their metabolites. These can have greater, equal, or lower toxicity than the parent compound, can accumulate under denitrifying conditions, and can negatively affect the denitrification process [3,7,19]. To verify this, it is necessary to perform research focused on metabolites and their behavior.

## 4. Conclusions

The presented research work has investigated the behavior of three pesticide compounds during the denitrification process. Adsorption of all tested pesticides to poplar wood shavings was in progress for the first seven days of the test and stagnated afterwards. The lowest adsorption extent was observed for alachlor; those for terbuthylazine and tebuconazole were higher. 

A high immediate adsorption of terbuthylazine to the poplar wood shavings was observed during the tests, which seems to be associated with the HgCl_2_ inhibitor. This behavior has not been observed before and should be analyzed in more detail in further studies. 

Next, our findings indicate that alachlor could be degraded in a biotic manner under denitrification conditions; however, the period of 28 days was not long enough to confirm this.

A comparison of the control treatment and the treatment with the tested pesticides demonstrated that alachlor, terbuthylazine, and tebuconazole do not have any inhibiting (negative) effect on the denitrification process; actually, they could have a stimulating (positive) effect on nitrate removal from water. Nevertheless, to obtain more robust results, we recommend employing more than four replications in every treatment or repeating the tests several times.

## Figures and Tables

**Table 1:** Methodology of the assays—composition of the samples.

Wood Shavings	Initial Liquid Medium, Solution in DIW		Treatment	Additional Reagents Added after 48 h
	c (NO_X_-N)	c (NaHCO_3_)		
25 g per bottle (COD > 100 mg L^−1^)	ST 30 mg L^−1^ LT 15 mg L^−1^	0.5 g L^−1^	C	0.1 mL pure methanol per L sample
			1	0.1 mL of the tested pesticide per L sample (conc. 1000 mg L^−1^ in pure methanol solution)
			2	0.1 mL of the tested pesticide per L sample (conc. 1000 mg L^−1^ in pure methanol solution) + 3.8 mL HgCl_2_ (123.5 mg L^−1^)

**Table 2:** Conditions at the end of the batch assays (average value ± standard deviation).

Parameter		pH (-)		DO (mg L^−1^)		NO_2_-N (mg L^−1^)		COD (mg L^−1^)	
Treatment		C	1	C	1	C	1	C	1
**TER**	**ST**	7.26 ± 0.03	7.20 ± 0.11	0.45 ± 0.05	0.33 ± 0.07	0.174 ± 0.090	0.227 ± 0.015	260 ± 33	260 ± 29
	**LT**	7.70 ± 0.13	7.78 ± 0.09	1.51 ± 0.79	2.50 ± 1.13	0.050 ± 0.004	0.076 ± 0.012	130 ± 18	120 ± 25
**ALA**	**ST**	7.51 ± 0.06	7.41 ± 0.17	0.39 ± 0.07	0.29 ± 0.02	3.596 ± 3.576	6.119 ± 2.165	290 ± 22	290 ± 41
	**LT**	7.71 ± 0.11	7.71 ± 0.14	1.89 ± 0.63	1.07 ± 0.27	0.120 ± 0.005	0.120 ± 0.006	110 ± 17	120 ± 19
**TEB**	**ST**	7.47 ± 0.05	7.48 ± 0.01	0.49 ± 0.08	0.35 ± 0.02	1.527 ± 1.421	6.528 ± 2.506	220 ± 0	270 ± 45
	**LT**	7.73 ± 0.08	7.77 ± 0.06	0.91 ± 0.22	1.89 ± 0.33	0.057 ± 0.022	0.062 ± 0.006	130 ± 11	100 ± 8

## Data Availability

Data are available upon request.
